# Advancements in Virtual Bioequivalence: A Systematic Review of Computational Methods and Regulatory Perspectives in the Pharmaceutical Industry

**DOI:** 10.3390/pharmaceutics16111414

**Published:** 2024-11-03

**Authors:** Nasser Alotaiq, Doni Dermawan

**Affiliations:** 1 Health Sciences Research Center, Imam Mohammad Ibn Saud Islamic University (IMSIU), Riyadh 11432, Saudi Arabia; 2 Department of Applied Biotechnology, Faculty of Chemistry, Warsaw University of Technology, 00-661 Warsaw, Poland; doni.dermawan.stud@pw.edu.pl

**Keywords:** pharmaceutical industry, physiologically based pharmacokinetic (PBPK) modelling, regulatory guidelines, virtual bioequivalence

## Abstract

Background/Objectives: The rise of virtual bioequivalence studies has transformed the pharmaceutical landscape, enabling more efficient drug development processes. This systematic review aims to explore advancements in physiologically based pharmacokinetic (PBPK) modeling, its regulatory implications, and its role in achieving virtual bioequivalence, particularly for complex drug formulations. Methods: We conducted a systematic review of clinical trials using computational methods, particularly PBPK modeling, to carry out bioequivalence assessments. Eligibility criteria are emphasized during in silico modeling and pharmacokinetic simulations. Comprehensive literature searches were performed across databases such as PubMed, Scopus, and the Cochrane Library. A search strategy using key terms and Boolean operators ensured that extensive coverage was achieved. We adhered to the PRISMA guidelines in regard to the study selection, data extraction, and quality assessment, focusing on key characteristics, methodologies, outcomes, and regulatory perspectives from the FDA and EMA. Results: Our findings indicate that PBPK modeling significantly enhances the prediction of pharmacokinetic profiles, optimizing dosing regimens, while minimizing the need for extensive clinical trials. Regulatory agencies have recognized this utility, with the FDA and EMA developing frameworks to integrate in silico methods into drug evaluations. However, challenges such as study heterogeneity and publication bias may limit the generalizability of the results. Conclusions: This review highlights the critical need for standardized protocols and robust regulatory guidelines to facilitate the integration of virtual bioequivalence methodologies into pharmaceutical practices. By embracing these advancements, the pharmaceutical industry can improve drug development efficiency and patient outcomes, paving the way for innovative therapeutic solutions. Continued research and adaptive regulatory frameworks will be essential in navigating this evolving field.

## 1. Introduction

Virtual bioequivalence (VBE) is an evolving pharmaceutical concept that leverages computational modeling and simulation techniques to assess the bioequivalence of generic drugs in regard to their reference or innovator counterparts [1,2]. Bioequivalence (BE) studies are a cornerstone of generic drug development, typically conducted to demonstrate that a generic drug exhibits pharmacokinetic (PK) and pharmacodynamic (PD) properties that are comparable to the original branded drug [3,4]. Traditionally, these studies require clinical trials involving human subjects, which can be costly, time-consuming, and often raise ethical concerns [5]. Such trials generally involve extensive sampling and PK testing to prove that the systemic drug exposure between the generic and branded drug does not differ significantly [6,7]. In contrast, virtual bioequivalence offers an alternative pathway, by employing advanced computational techniques to predict the bioequivalence of generic drugs without the need for large-scale clinical trials [8]. This approach involves developing and applying mathematical models that simulate the drug absorption, distribution, metabolism, and excretion (ADME) processes in humans. Physiologically based pharmacokinetic (PBPK) modeling, computational fluid dynamics (CFD), and in silico dissolution modeling are key tools in this domain [9,10]. These models integrate drug properties (e.g., solubility, permeability), formulation characteristics, and physiological parameters, providing a mechanistic framework that helps predict how drugs behave in the human body [11,12]. PBPK models, in particular, are gaining prominence due to their ability to simulate complex physiological processes with high predictive power. They can replace empirical approaches, offering a more nuanced understanding of drug absorption and bioavailability [13,14]. These models are increasingly used in drug discovery, development, and regulatory submissions, due to their capacity to integrate a wide array of data, including genetic variability and individual patient characteristics, thus enhancing prediction accuracy [15].

Regulatory agencies, such as the U.S. Food and Drug Administration (FDA), the European Medicines Agency (EMA), and other global regulatory bodies, have recognized the potential of virtual bioequivalence. These agencies have begun to introduce guidelines that support the use of modeling and simulation in bioequivalence studies [16]. The FDA, for instance, has published guidance documents encouraging the application of PBPK models in regulatory submissions, particularly for complex, generic drug products. Similarly, the EMA has issued draft guidelines acknowledging the role of in silico methods in demonstrating bioequivalence, especially when conventional approaches may be infeasible due to ethical or practical constraints [17,18,19]. The acceptance of virtual bioequivalence by regulatory bodies relies heavily on the validation of computational models and the availability of accurate, comprehensive data inputs. Model validation is essential to ensure that the virtual predictions align closely with the in vivo results [1]. The reliability of virtual bioequivalence studies also hinges on the quality of data used to populate these models, such as precise drug physicochemical properties, human physiology parameters, and clinical trial data [2]. While virtual bioequivalence holds immense promise, several challenges must be addressed. One of the critical challenges is the validation of computational models across a wide range of drugs and formulations. Ensuring that the in silico results correlate strongly with the clinical outcomes is essential in gaining regulatory acceptance [20]. Additionally, the lack of standardized methodologies and limited regulatory approval in certain regions can hinder the broader adoption of virtual bioequivalence [21,22]. Furthermore, there is a need for ongoing research to establish robust correlations between virtual bioequivalence models and in vivo outcomes, particularly in regard to complex drug formulations, such as biologics and combination products [23]. Despite these challenges, the future of virtual bioequivalence looks promising. Emerging technologies, such as artificial intelligence (AI) and machine learning (ML), are poised to further enhance the predictive capabilities of PBPK and other computational models. These technologies can help optimize model performance by learning from large datasets, identifying patterns, and refining simulations, to improve prediction accuracy [24,25]. Integrating AI and ML with virtual bioequivalence offers the potential for the creation of personalized medicine, where models can be tailored to individual patient characteristics, enabling more precise predictions of drug behavior.

This systematic review aims to assess the current state of virtual bioequivalence in the pharmaceutical industry, by examining the computational methods and regulatory perspectives involved. The review details the methodologies and tools used in virtual bioequivalence, highlights successful applications, and discusses the advantages and challenges to this approach. Additionally, it explores future perspectives on integrating virtual bioequivalence with emerging technologies, such as AI and ML. The sources for this review included major databases, such as PubMed, Scopus, Web of Science, and the Cochrane Library, as well as regulatory websites for organizations like the FDA and EMA.

## 2. Materials and Methods

### 2.1. Eligibility Criteria

This review focused exclusively on clinical trials involving pharmaceutical drugs and bioequivalence studies, specifically those evaluating computational methods for virtual bioequivalence. The inclusion criteria were limited to studies using in silico modeling, pharmacokinetics/pharmacodynamics simulations, and other digital platforms for virtual bioequivalence assessments. Comparisons were made between these computational methods and traditional clinical bioequivalence trials, where available, and among different computational tools. The primary outcomes of interest were these methods’ efficacy, accuracy, and reliability, along with their compliance with regulatory standards set by agencies such as the FDA and EMA. Systematic reviews, narrative reviews, and studies lacking a focus on computational methods or regulatory perspectives were excluded.

### 2.2. Information Sources

To ensure comprehensive coverage of the relevant literature, the review utilized several key databases: PubMed, Scopus, Web of Science, Embase, and the Cochrane Library. These databases provided access to peer-reviewed articles, conference proceedings, and systematic reviews. Additionally, Google Scholar was used to identify grey literature, including theses and reports not indexed in traditional databases. Regulatory agency websites, including those by the FDA and EMA, were also consulted for guidelines and standards related to virtual bioequivalence. The review focused on studies published from 2014 to 2024, to capture recent advancements and trends. Only studies published in English were included, to ensure the accessibility and comprehensibility of the reviewed literature.

### 2.3. Search Strategy

A comprehensive search strategy was employed, using a combination of key terms related to virtual bioequivalence and computational methods. The terms included “Virtual Bioequivalence”, “In Silico Bioequivalence”, “Computational Modelling”, “Simulation”, “Physiologically Based Pharmacokinetic (PBPK) Modelling”, “Computational Fluid Dynamics (CFD)”, “In Silico Dissolution Modelling”, “Generic Drugs”, “Innovator Drugs”, “Bioequivalence Assessment”, “Pharmaceutical Industry”, “Drug Development”, and “Regulatory Guidelines.” Boolean operators (AND, OR), truncation, and MeSH (Medical Subject Headings) terms were applied to refine the search and ensure the comprehensiveness of the coverage.

### 2.4. Study Selection

The study selection process involved an initial screening of titles and abstracts to determine the relevance, based on the inclusion criteria. Studies that met these criteria proceeded to a full-text review. The Preferred Reporting Items for Systematic Reviews and Meta-Analyses (PRISMA) flow diagram was used to document the number of studies at each stage of the review process, including identification, screening, eligibility, and final inclusion. This approach provided a clear and transparent overview of the selection process and ensured that only relevant studies were included.

### 2.5. Data Extraction

Data extraction was performed using a standardized form, to ensure consistency across the studies. The key data collected included study characteristics, such as the author, publication year, journal, and country. Details on the computational methods used, including PBPK modeling, CFD, and in silico dissolution modeling, were recorded. The regulatory frameworks considered in the studies were also documented, focusing on guidelines and standards from regulatory bodies like the FDA and EMA. Key outcomes related to accuracy, regulatory compliance, and computational challenges were extracted, along with assessments of the study quality and potential biases.

## 3. Results

### 3.1. Systematic Literature Search and Study Selection Process

**Figure 1:**
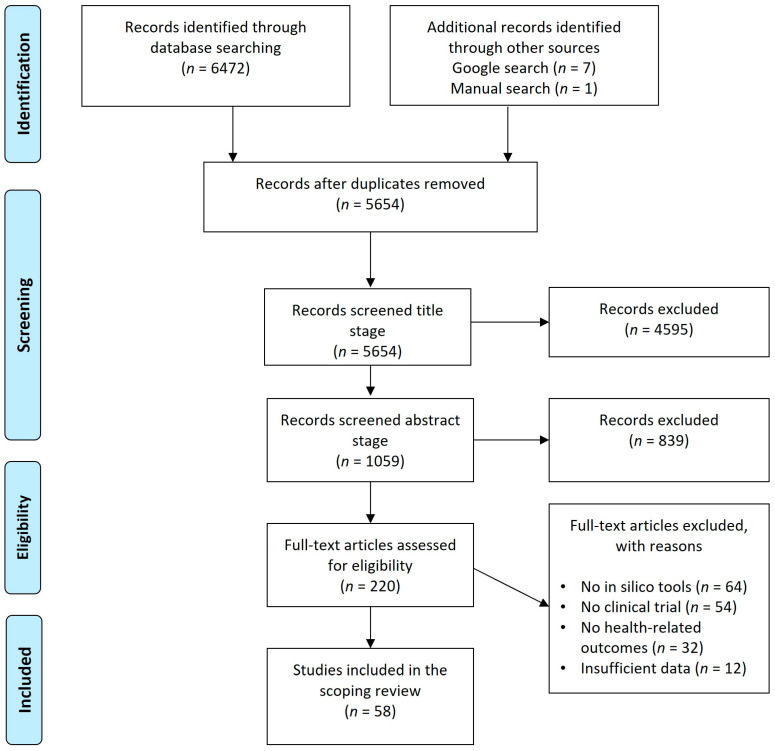
PRISMA (Preferred Reporting Items for Systematic Reviews and Meta-Analyses) flow diagram illustrating the study selection process, from identification through to inclusion, outlining the number of records screened, excluded, and included in the final scoping review.

A systematic literature search was conducted to identify relevant studies exploring virtual bioequivalence through in silico tools. The identification phase began with a comprehensive search across the databases (PubMed, Scopus, Web of Science, and the Cochrane Library), resulting in 6472 records (Figure 1). Additionally, eight records were found through external sources, including a Google search and manual searches of journal by hand. After the removal of duplicates, 5654 records remained for screening. During the screening phase, a title-based screening of the 5654 records was performed. This stage resulted in the exclusion of 4595 records that were deemed irrelevant based on their titles, leaving 1059 records to progress to abstract screening. Upon reviewing the abstracts, 839 records were further excluded for not meeting the inclusion criteria. The remaining 220 records proceeded to a full-text assessment during the eligibility phase. At the full-text screening stage, 162 articles were excluded, each for specific reasons: 64 studies did not involve in silico tools, 54 articles lacked any connection to clinical trials, 32 articles were unrelated to health outcomes, and 12 articles failed to provide sufficient data. This rigorous assessment led to the inclusion of 58 studies in the final scoping review.

The results of this review demonstrate that despite the initial large number of records identified, only a small fraction of studies met the stringent inclusion criteria for assessing virtual bioequivalence. The high rate of exclusion highlights the specificity of the focus on in silico methodologies and the limited availability of studies directly comparing computational tools with traditional clinical bioequivalence trials. One significant finding was the exclusion of a substantial number of studies that did not utilize in silico tools (64 records), which underscores the emerging, but still nascent nature of the use of computational methods in bioequivalence assessment. Furthermore, the exclusion of studies not involving clinical trials (54 records) points to the challenge of integrating virtual bioequivalence tools into clinical research, as many studies may focus solely on theoretical modeling without validation in real-world clinical settings. The exclusion of studies due to insufficient data (12 records) also emphasizes the importance of robust data reporting and transparency in in silico research. For virtual bioequivalence to gain widespread regulatory acceptance, comprehensive data and validation will be crucial. In addition to the primary studies included in this scoping review, regulatory guidelines and frameworks regarding virtual bioequivalence were retrieved from major regulatory bodies, notably the FDA and EMA. Both organizations have established guidelines for bioequivalence assessments, but specific provisions related to the use of in silico tools in these assessments are still evolving. Table 1 summarizes the studies included in the scoping review. The main findings from each study are available in the Supplementary Materials S1.

### 3.2. Utilization of In Silico Modeling Tools in Virtual Bioequivalence Studies

**Figure 2:**
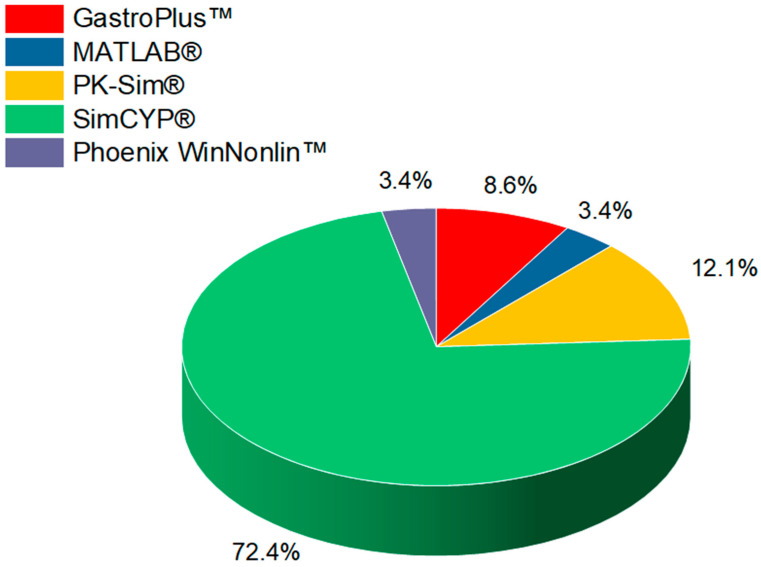
Pie chart depicting the distribution of in silico modeling tools used in virtual bioequivalence studies, as identified in the systematic review. The chart highlights the percentage utilization of each tool, including SimCYP^®^, PK-Sim^®^, GastroPlus™, Phoenix WinNonlin™, and MATLAB^®^.

The systematic review revealed that various in silico tools have been employed to assess virtual bioequivalence, with SimCYP^®^ emerging as the most widely used, appearing in 42 out of the 58 selected studies (72.4%), as presented in Figure 2. SimCYP^®^ is a sophisticated PBPK modeling platform, renowned for its ability to simulate the complex processes of drug ADME within diverse virtual populations. Its strength lies in its detailed modeling of drug–drug interactions, particularly those involving cytochrome P450 enzymes and key transporter proteins [84]. This capability allows researchers to explore how genetic variability, disease conditions, and demographic factors, such as age and ethnicity, influence drug pharmacokinetics and dynamics [85]. One of the main advantages of SimCYP^®^ is its predictive power in modeling drug interactions. It can accurately simulate the impact of co-administered medications or dietary components on drug clearance and bioavailability. It has been proven to be especially useful in predicting how CYP enzyme inhibitors or inducers modify drug exposure, providing critical insights into the risks of toxicity or therapeutic failure [86,87]. This is particularly valuable for bioequivalence studies, where the goal is to determine whether a generic drug performs similarly to an innovator drug in various patient populations without conducting large-scale clinical trials. Moreover, the ability of SimCYP^®^ to integrate population variability makes it a favored choice for predicting bioequivalence outcomes across different subpopulations, such as those with liver or kidney impairment, the elderly, or individuals with specific genetic polymorphisms. These simulations reduce the need for multiple clinical trials involving distinct patient groups, making virtual bioequivalence studies more efficient, faster, and more cost effective [88,89]. SimCYP^®^’s broad application in bioequivalence studies highlights its versatility and regulatory acceptance. It has been widely used to support regulatory submissions to agencies like the FDA and EMA, as it complies with their requirements for bioequivalence assessments, particularly in cases involving complex drug–drug interactions or populations at the extremes of pharmacokinetic variability [90,91]. Thus, SimCYP^®^ continues to play a critical role in virtual bioequivalence assessments, contributing to a more streamlined drug development process.

Following SimCYP^®^, PK-Sim^®^ was the next most commonly used in silico tool, appearing in seven studies (12.1%). PK-Sim^®^ is another widely adopted PBPK modeling platform, known for its detailed representations of whole-body physiology and customizable drug kinetic profiles. Unlike SimCYP^®^, which excels in terms of virtual population simulations and drug–drug interaction predictions, PK-Sim^®^ has a notable strength in its integration with MoBi^®^. This system’s biology modeling tool allows for advanced mechanistic and dynamic modeling. This capability makes PK-Sim^®^ particularly effective in studying complex drug interactions, multi-compartmental pharmacokinetics, and situations where drugs demonstrate non-linear kinetics [13,92]. One key advantage of PK-Sim^®^ is its flexibility during simulated physiological scenarios beyond typical bioequivalence conditions. For instance, it has been employed to simulate pharmacokinetic profiles in special populations, such as pediatric, elderly, or renal-impaired patients, where clinical data may be sparse or difficult to obtain. This strength is particularly relevant in regulatory settings, where simulations can be used to predict pharmacokinetic behaviors in conditions that are difficult to test using traditional clinical trials [93]. Additionally, PK-Sim^®^’s capacity to model multi-compartment systems, where drugs may exert effects at multiple sites, encounter varying enzyme activities, or exhibit complex absorption and clearance patterns, further demonstrates its utility in bioequivalence studies that involve non-linear kinetics or intricate pharmacological behaviors [94].

GastroPlus™, utilized in five studies (8.6%), stands out as a specialized software, primarily focusing on predicting drug dissolution, absorption, and pharmacokinetics within the gastrointestinal tract. GastroPlus™ integrates PBPK and in silico dissolution models to simulate how drug formulations behave within the human digestive system, making it particularly useful for evaluating oral bioavailability and the influence of various formulation factors on drug performance. This tool has been employed to simulate bioequivalence for modified-release formulations or drugs with low solubility, where gastrointestinal transit times and dissolution play critical roles in pharmacokinetics [95,96]. Two studies (3.4%) used MATLAB for pharmacokinetic modeling. While MATLAB is not a dedicated PBPK software, it offers advanced computational capabilities and flexibility for creating custom pharmacokinetic and pharmacodynamic (PK/PD) models. MATLAB is frequently employed for exploratory analysis, custom data fitting, and simulations of drug kinetics in scenarios where commercial software might not provide the necessary specificity. For virtual bioequivalence, MATLAB can be used to build highly tailored models that simulate drug behavior under varying conditions or in populations not easily represented by standard tools [48,49]. In addition to the use of GastroPlus™ and MATLAB, Phoenix WinNonlin™ was employed in two studies (3.4%) for pharmacokinetic and bioequivalence modeling. Phoenix WinNonlin™ is recognized for its robust statistical and compartmental modeling capabilities, particularly in non-compartmental analysis (NCA), a key method for bioequivalence assessments. This software is especially beneficial for analyzing and interpreting PK data in regulatory submissions, offering validated modules that meet industry standards for PK/PD, and bioequivalence analyses. In virtual bioequivalence, Phoenix WinNonlin™ allows for precise data fitting and the assessment of drug concentration–time profiles, which is valuable for determining equivalence between formulations [62,63,97].

### 3.3. Overview of Disease Type Distribution in Virtual Bioequivalence Studies

**Figure 3:**
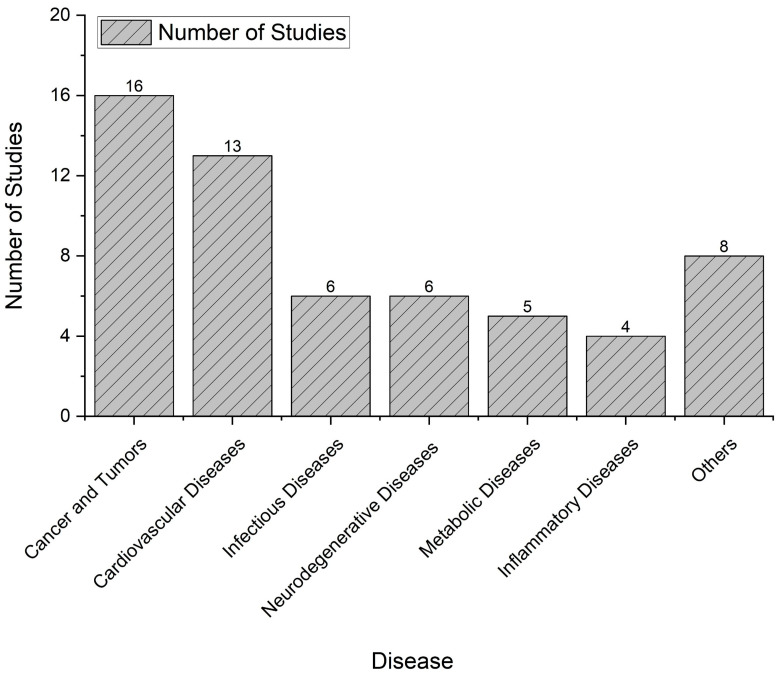
Prevalence of disease types in virtual bioequivalence research (2014–2024). This figure illustrates the distribution of various disease types investigated in studies utilizing virtual bioequivalence over the past decade.

This systematic review, conducted on the application of virtual bioequivalence from 2014 to 2024, reveals distinct trends in regard to disease type prevalence among the studies analyzed. Cancer and tumors emerged as the most extensively researched categories, accounting for a total of 16 studies (Figure 3). This significant focus underscores the critical need for effective therapeutic interventions and the potential role of virtual bioequivalence in optimizing drug formulations for oncology. After cancer, cardiovascular diseases was the second most studied category, with 13 studies identified. This emphasis highlights the ongoing challenges in managing cardiovascular health and the potential for virtual bioequivalence methodologies to contribute to the development of safer and more effective cardiovascular therapies. Infectious diseases and neurodegenerative diseases each comprised six studies. The interest in infectious diseases reflects global health priorities and the urgent need for innovative approaches to combating various pathogens. Similarly, the focus on neurodegenerative diseases indicates a growing recognition of their complex treatment landscapes and the necessity for advanced computational strategies to enhance drug development in this area. Additionally, metabolic diseases were addressed in five studies, while inflammatory diseases were covered in four studies. The relatively lower number of studies in these categories suggests that they are potential areas for further exploration within the context of virtual bioequivalence, as they represent significant public health concerns. Lastly, a collection of eight studies examined various other diseases, illustrating the versatility and applicability of virtual bioequivalence across a broad range of medical conditions. This diversity in the research underscores the potential for virtual bioequivalence to inform drug development and optimization across multiple therapeutic areas.

The emerging field of virtual bioequivalence studies in the context of cancer and tumor treatment has witnessed significant advancements in recent years, largely facilitated by the application of PBPK modeling. Various studies have explored the pharmacokinetics of specific anticancer drugs, enhancing our understanding of their interactions, absorption characteristics, and overall therapeutic efficacy. For instance, Yu et al. (2017) developed a PBPK model for palbociclib, an anticancer agent, predicting that moderate CYP3A inhibitors could elevate the AUC by approximately 40%, while inducers might reduce it by a similar margin. This model demonstrated a strong correlation between the predicted and observed data, establishing a reliable tool for assessing drug interactions [26]. Similarly, Chen et al. (2017) utilized PBPK modeling to analyze gefitinib, revealing a 39% reduction in the AUC for CYP2D6 ultrarapid metabolizers compared to extensive metabolizers. Importantly, the reduced exposure remained above the inhibitory concentration for EGFR mutations in non-small-cell lung cancer, indicating that the drug’s efficacy would likely remain intact in clinical settings [39]. In another study, Gajewska et al. (2020) assessed the oral absorption of alpelisib through a PBPK model, successfully simulating bioequivalence between clinical and commercial formulations under varying conditions. The predictive capacity of the model was validated with errors in the plasma Cmax and an AUC below 30%, making it a critical asset for formulation development. Moreover, studies involving the prediction of DDIs have highlighted the utility of PBPK modeling [40]. Freise et al. (2017) demonstrated that the pharmacokinetics of venetoclax could be significantly altered by CYP3A inhibitors and inducers, recommending necessary dose adjustments to maintain therapeutic levels [61]. Similarly, Yamazaki et al. (2015) validated a PBPK model for crizotinib, affirming that the results from single-dose studies could guide multiple-dose scenarios, thereby enhancing the efficiency of drug administration strategies [56]. Recent studies have extended these methodologies to pediatric populations, as illustrated by Morcos et al. (2023), who employed model-informed approaches to support dosing recommendations for copanlisib in children with relapsed/refractory solid tumors [71].

The exploration of virtual bioequivalence studies has gained considerable traction in the context of cardiovascular diseases, emerging as the second most researched area after cancer and tumor studies. For instance, Donovan et al. (2018) developed a PBPK model to predict the plasma and brain concentrations of bumetanide in different populations, highlighting the model’s limited applicability in neonates, due to insufficient metabolic and transport parameter data [41]. Similarly, Dennison et al. (2017) assessed the bioavailability of fixed-dose combination, orally disintegrating tablets containing amlodipine and atorvastatin. Their findings indicated no significant differences in the bioavailability between the single and fixed-dose formulations, although atorvastatin showed increased exposure in fed subjects, suggesting the influence of gut transit dynamics [59]. Furthermore, Tsamandouras et al. (2015) created a population PBPK model for simvastatin, successfully predicting plasma concentrations and the effects of genetic polymorphisms on drug interactions and toxicity [73]. Rhee et al. (2018) focused on potential drug–drug interactions involving fimasartan, amlodipine, and hydrochlorothiazide, finding no significant interactions that were consistent with clinical observations [76]. Additionally, Chen et al. (2022) investigated salvianolic acid A using a PBPK model that uncovered challenges related to dose proportionality in pharmacokinetics, specifically the saturation of transport mechanisms at higher doses [82]. Lastly, Kaur et al. (2020) examined the oral absorption of irbesartan, utilizing biorelevant dissolution testing and PBPK modeling to enhance the accuracy of plasma exposure predictions [83]. These studies collectively underscore the growing importance of virtual bioequivalence methodologies in cardiovascular pharmacology. They facilitate more precise dosing recommendations and improve the understanding of drug behaviors across different populations and formulations.

Research into virtual bioequivalence for infectious diseases, including bacterial, fungal, and viral infections, has positioned itself as a significant area of study, ranking third after cardiovascular and cancer-related investigations. This field has benefited from advanced in silico tools, particularly PBPK modeling, which has been pivotal in understanding drug interactions and optimizing dosing regimens for various antimicrobial agents. For example, Yee et al. (2020) utilized PBPK modeling to analyze doravirine, revealing that co-administration with rifabutin led to a substantial decrease in doravirine exposure. Their findings necessitated a dosing adjustment from 100 mg once daily to 100 mg twice daily to achieve the desired pharmacokinetic profile, confirming the accuracy of the model predictions through subsequent clinical trials [33]. In the antifungal domain, Zane et al. (2014) developed a PBPK model for voriconazole, which initially overestimated oral bioavailability in pediatric patients, but improved significantly upon accounting for intestinal first-pass metabolism. This highlights the need for tailored pharmacokinetic assessments in different age groups [36]. Similarly, Bergagnini-Kolev et al. (2023) examined inhaled itraconazole and its potential in terms of drug–drug interactions, predicting minimal risk when co-administered with midazolam due to low systemic itraconazole exposure, underscoring the safety of this formulation for treating pulmonary fungal infections [43]. Additionally, Stader et al. (2021) focused on bictegravir, a drug used in HIV treatment, and found that aging did not significantly affect its pharmacokinetics, indicating that no dose adjustment is needed for elderly individuals without severe comorbidities [49]. Salerno et al. (2017) created a PBPK model for solithromycin, effectively predicting plasma and epithelial lining fluid concentrations, with simulations showing high efficacy in achieving target exposure ratios in patients with community-acquired bacterial pneumonia [74].

### 3.4. Regulatory Perspectives on Virtual Bioequivalence in the Pharmaceutical Industry

The rise of virtual bioequivalence studies in drug development, particularly concerning cardiovascular diseases, has been increasingly recognized by regulatory agencies, such as the FDA and EMA. As the pharmaceutical landscape evolves, these agencies have begun to incorporate advanced computational modeling and simulation techniques to enhance the evaluation of bioequivalence, ensuring the safety and efficacy of generic formulations. The FDA has developed a framework for the use of in silico methods in pharmacokinetic studies, particularly under its Guidance for Industry: Bioavailability and Bioequivalence Studies for Orally Administered Drug Products—General Considerations. This guidance acknowledges the role of PBPK modeling in predicting the pharmacokinetics of drugs in various populations, including special populations, such as pediatrics and the elderly. The FDA encourages the use of PBPK models to simulate drug ADME, which can significantly reduce the need for extensive clinical trials, particularly in cases where conducting such trials would be ethically or logistically challenging. For instance, in the studies highlighted in our systematic review, researchers like Dennison et al. (2017) and Kaur et al. (2020) employed PBPK modeling to predict the bioavailability and oral absorption profiles for combination therapies and individual drugs, respectively [59,83]. Their findings align with the FDA’s recommendations, demonstrating how in silico approaches can provide reliable data that can be used to inform dosing regimens and improve therapeutic outcomes without the need for exhaustive clinical testing. The EMA similarly recognizes the potential of virtual bioequivalence studies in its Guideline on the Investigation of Bioequivalence. The EMA encourages the use of modeling and simulation as part of the drug development process, particularly for complex formulations, such as fixed-dose combinations and modified-release products. The agency emphasizes that robust PBPK models can facilitate a better understanding of the impact of physiological variability and drug–drug interactions on bioavailability. In the context of the studies reviewed, Rhee et al. (2018) successfully employed PBPK modeling to assess the drug–drug interaction potential of fimasartan, amlodipine, and hydrochlorothiazide [76]. This study exemplifies the EMA’s approach, as it combined data from the literature, in silico tools, and in vitro studies, to build a validated model that accurately predicted pharmacokinetic outcomes in healthy subjects. Such predictive capabilities not only streamline the development process, but also ensure compliance with regulatory standards. Moreover, the review points out the critical need for regulatory bodies to provide clear frameworks that support the integration of these advanced methodologies into standard bioequivalence assessments. As evidenced by ongoing studies and their implications for clinical practice, establishing a robust regulatory framework that accommodates virtual bioequivalence can lead to more efficient drug development processes, reduced costs, and improved patient outcomes.

## 4. Discussion

### 4.1. Significance of the Systematic Review Results and Its Correlation with Other Studies

The integration of virtual bioequivalence methodologies, particularly through PBPK modeling, has significant implications for the pharmaceutical industry. By leveraging these advanced computational techniques, companies can streamline the drug development process, leading to reduced time and costs associated with bringing new medications to market [98,99,100]. One of the most notable impacts is enhanced efficiency in predicting pharmacokinetic profiles. PBPK modeling allows for the simulation of drug ADME in diverse populations, including vulnerable groups, such as children and the elderly [101]. This capability not only optimizes dosing regimens, but also minimizes the need for extensive clinical trials, which can be logistically challenging and ethically complex. For example, the use of PBPK models has been shown to effectively predict bioavailability in complex drug formulations, facilitating faster regulatory approval and more timely patient access to therapies. The findings from this systematic review underscore the growing importance of virtual bioequivalence studies in the pharmaceutical industry, particularly in optimizing the drug development process. This systematic review highlights several key insights that correlate with the existing literature and reflect the shifting paradigms in terms of the regulatory perspectives on bioequivalence. This review highlights that the incorporation of PBPK modeling and simulation techniques substantially improves the efficiency of drug development processes. For example, several research studies illustrate that PBPK models can reliably predict the pharmacokinetic profiles of drugs, enabling researchers to optimize dosing regimens without the need for extensive clinical trials [102,103]. This finding reinforces our review’s assertion that virtual bioequivalence can effectively streamline drug approval processes, while ensuring adherence to safety and efficacy standards. Moreover, the alignment between our review’s findings and the evolving regulatory perspectives is significant. Both the FDA and EMA are increasingly acknowledging the value of in silico methods, as evidenced by the comprehensive frameworks they are developing for the application of PBPK modeling. For instance, several studies discuss the FDA’s proactive approach to facilitating the integration of computational modeling into drug evaluations, emphasizing its importance in assessing complex formulations [104,105]. This underscores our review’s call for robust regulatory guidelines to accommodate virtual bioequivalence methodologies, ultimately fostering innovation within the pharmaceutical landscape. Additionally, the integration of virtual bioequivalence with real-world evidence (RWE) is a significant development. A study advocates for combining RWE with computational modeling to validate bioequivalence claims further, thereby addressing the variability observed in clinical settings [106]. This perspective reinforces the importance of our review’s findings, as it suggests a holistic approach to drug development that incorporates various data sources to optimize therapeutic strategies.

### 4.2. Limitations of This Systematic Review

While this systematic review provides valuable insights, several limitations must be acknowledged. One key limitation is the heterogeneity among the included studies. Variability in the study design, populations, and methodologies can impact the comparability of the results. For instance, differences in how PBPK modeling is implemented across the studies may lead to inconsistencies in the findings. This heterogeneity could restrict the generalizability of our conclusions and underscores the necessity for standardized protocols in virtual bioequivalence research. Another limitation pertains to the potential for publication bias. Studies that report successful outcomes for virtual bioequivalence are often more likely to be published, while those with negative or inconclusive results may remain unpublished. This bias can distort the overall understanding of the efficacy and reliability of virtual bioequivalence methodologies, a concern also highlighted in similar systematic reviews. Addressing publication bias requires greater transparency in research reporting, including the publication of negative results. Additionally, the search strategy employed in this systematic review may present limitations. Although comprehensive, it is possible that relevant studies were overlooked due to the inclusion criteria. For instance, studies published in languages other than English or less prominent journals may not have been captured, potentially omitting valuable data and perspectives in the field. Finally, the rapid evolution of regulatory frameworks concerning virtual bioequivalence poses another challenge. As the FDA and EMA continue to refine their guidelines, the findings of this review may need to be revisited and updated regularly. This dynamic regulatory landscape implies that the implications of this systematic review could change over time, emphasizing the importance of ongoing research and monitoring in this area.

## 5. Conclusions

In conclusion, this systematic review underscores the significant advancements in virtual bioequivalence, particularly through the adoption of computational methods, such as PBPK modeling. These innovative approaches enhance the efficiency and reliability of drug development processes, allowing for more accurate predictions of pharmacokinetic profiles and optimized dosing regimens. The growing recognition of the value of in silico methods by regulatory agencies, like the FDA and EMA, further solidifies their importance in evaluating complex drug formulations, while ensuring safety and efficacy standards. Moreover, this review highlights the critical need for robust regulatory guidelines to support the integration of virtual bioequivalence methodologies into standard practices. This support will facilitate innovation within the pharmaceutical landscape, ultimately improving patient outcomes and streamlining drug approval processes. While acknowledging the limitations to the current body of research, including study heterogeneity and potential publication bias, the findings point to a promising future for virtual bioequivalence. Continued efforts in regard to standardization, transparency in research reporting, and adaptive regulatory frameworks, will be essential to harness the full potential of these computational techniques in drug development.

## Figures and Tables

**Table 1:** A summary of the studies included in the scoping review, outlining the pharmaceutical drugs involved, therapeutic indications, in silico tools used, and the key findings related to bioequivalence.

Study; Year	Database	Pharmaceutical Drug(s)	Drug(s) Indication Class	In Silico Tool(s)
Yu, Y. et al. [26], 2017	PubMed	Palbociclib	Anticancer	SimCYP^®^ version 14
Cho, CK. et al. [27], 2021	PubMed	Tamsulosin	Benign prostatic hyperplasia (BPH)	SimCYP^®^
Chen, G. et al. [28], 2023	PubMed	Maribavir	Anti-cytomegalovirus	SimCYP^®^ version 17
Kim Y.H. et al. [29], 2021	PubMed	Celecoxib	NSAIDs	PK-Sim^®^ version 7.2
Fendt, R. et al. [30], 2021	PubMed	Caffeine	Neurostimulant	PK-Sim^®^ version 8.0
Watanabe, A. et al. [31], 2021	PubMed	Esaxerenone	Antimineralocorticoid	SimCYP^®^, version 17
Ou, Y. et al. [32], 2018	PubMed	Oprozomib	Antitumor	SimCYP^®^ version 13.2
Yee, K.L. et al. [33], 2020	PubMed	Doravirine	Antiretroviral	SimCYP^®^ version 17
Jo, H. et al. [34], 2021	PubMed	Dapagliflozin	Antidiabetic	SimCYP^®^ version 18
Posada, M. et al. [35], 2017	PubMed	Baricitinib	Antirheumatic	SimCYP^®^ version 13.2
Zane, N.R. et al. [36], 2014	PubMed	Voriconazole	Antifungal	SimCYP^®^ Paediatric
Lang, J. et al. [37], 2020	PubMed	Ivabradine	Antianginal and anti-ischemic	SimCYP^®^ version 18
Hanke, N. et al. [38], 2017	PubMed	Zoptarelin doxorubicin	Anticancer	PK-Sim^®^ and MoBi^®^
Chen, Y. et al. [39], 2017	PubMed	Gefitinib	Anticancer	SimCYP^®^ version 14
Gajewska, M. et al. [40], 2020	PubMed	Alpelisib	Anticancer	GastroPlus™ version 9.6
Donovan, M.D. et al. [41], 2018	PubMed	Bumetanide	Cardiovascular disease	SimCYP^®^ version 14
Fu, Q. et al. [42], 2021	PubMed	Lenabasum	Anti-inflammatory	SimCYP^®^ version 19
Bergagnini-Kolev, M. et al. [43], 2023	PubMed	Itraconazole	Antifungal	SimCYP^®^
Nakamaru, Y. et al. [44], 2015	PubMed	Teneligliptin	Antidiabetic	SimCYP^®^
Thompson, E.J. et al. [45], 2024	PubMed	Pantoprazole	GERD	PK-Sim^®^ version 10.0
Wang, H.Y. et al. [46], 2016	PubMed	Midazolam	Hypnotic sedative	SimCYP^®^ version 13
Callegari, E. et al. [47], 2021	PubMed	Ertugliflozin	Antidiabetic	SimCYP^®^ version 15
Nakamura, T. et al. [48], 2018	PubMed	Tamoxifen	Anticancer	MATLAB version 8.0.0.783 (R2012b)
Stader, F. et al. [49], 2021	PubMed	Bictegravir	Antiretroviral	Matlab 2017a
Yang, R. et al. [50], 2024	PubMed	Omeprazole	GERD	SimCYP^®^
Ojala, K. et al. [51], 2020	PubMed	ODM-204	Anticancer	GastroPlus version 9.7
Agyemang, A. et al. [52], 2021	PubMed	Acumapimod	Anti-inflammatory	SimCYP^®^ version 16.1
Chen, Y. et al. [53], 2014	PubMed	Amiodarone	Anti-arrhythmic	SimCYP^®^ version 12
Litou, C. et al. [54], 2019	PubMed	Aprepitant	Antiemetic	SimCYP^®^ version 16.1
Einolf, H.J. et al. [55], 2017	PubMed	Sonidegib	Anticancer	SimCYP^®^ version 13.2
Yamazaki, S. et al. [56], 2015	PubMed	Crizotinib	Anticancer	SimCYP^®^ version 13.1
Riddell, K. et al. [57], 2020	PubMed	Molibresib	Anticancer	SimCYP^®^ version 14
Purohit, V. et al. [58], 2024	PubMed	Tofacitinib	Antirheumatic	SimCYP^®^ version 20
Dennison, T.J. et al. [59], 2017	PubMed	Amlodipine and atorvastatin	Cardiovascular disease	SimCYP^®^ version 14
Knöchel, J. et al. [60], 2024	PubMed	Midazolam	Hypnotic sedative	SimCYP^®^ version 20
Freise, K.J. et al. [61], 2017	PubMed	Venetoclax	Anticancer	SimCYP^®^ version 14
Zhang, M. et al. [62], 2024	PubMed	Rivaroxaban, ticagrelor, and PB-201	Cardiovascular disease	SimCYP^®^ version 20
Ding, S. et al. [63], 2020	PubMed	Rivaroxaban	Cardiovascular disease	Phoenix WinNonlin™ version 7.0
Wang, J. et al. [64], 2021	PubMed	Ticagrelor	Ticagrelor	Phoenix WinNonlin™ version 6.3
Boetsch, C. et al. [65], 2016	Web of Science	Bitopertin	Neurodegenerative disease	GastroPlus™
Katsube, T. et al. [66], 2020	Web of Science	Lusutrombopag	Thrombocytopenia	SimCYP^®^ version 14
Andreas, C.J. et al. [67], 2017	Web of Science	Zolpidem	Hypnotic sedative	Simcyp^®^ and GastroPlus™
Post, T.M. et al. [68], 2016	Web of Science	Nomegestrol acetate	Hormone therapy	PK-Sim^®^
Li, J. et al. [69], 2020	Web of Science	Eliglustat	Gaucher’s disease	SimCYP^®^ version 13
Samant, T.S. et al. [70], 2018	Web of Science	Ribociclib	Anticancer	SimCYP^®^ version 13
Morcos, P.N. et al. [71], 2023	Web of Science	Copanlisib	Anticancer	PK-Sim^®^
Traver, E. et al. [72], 2024	Web of Science	Leriglitazone	CNS diseases	SimCYP^®^ version 17
Tsamandouras, N. et al. [73], 2015	Web of Science	Simvastatin	Cardiovascular disease	SimCYP^®^ version 13
Salerno, S. et al. [74], 2017	Cochrane Library	Solithromycin	Antibiotic	PK-Sim and MoBi version 6.2
Venuto, C. et al. [75], 2020	Cochrane Library	Nilotinib	Anticancer	SimCYP^®^
Rhee, S.J. et al. [76], 2018	Cochrane Library	Fimasartan, amlodipine, and hydrochlorothiazide	Cardiovascular disease	SimCYP^®^ version 15
Hwang, S. et al. [77], 2024	Cochrane Library	Methotrexate	Antirheumatic	SimCYP^®^ version 21
Samant, T.S. et al. [78], 2020	Cochrane Library	Ribociclib	Anticancer	SimCYP^®^ version 18
Chen, B. et al. [79], 2022	Cochrane Library	Acalabrutinib	Anticancer	SimCYP^®^ version 19
Djebli, N. et al. [80], 2015	Cochrane Library	Clopidogrel	Antiplatelet	SimCYP^®^ version 10.2
Xiao, Q. et al. [81], 2015	Cochrane Library	Repaglinide and pioglitazone	Antidiabetic	SimCYP^®^ version 14.1
Chen, J. et al. [82], 2022	Cochrane Library	Salvianolic acid A	Cardiovascular disease	GastroPlus^®^ Version 9.8
Kaur, N. et al. [83], 2020	Cochrane Library	Irbesartan	Cardiovascular disease	GastroPlus™

## Data Availability

The original contributions presented in this study are included in the article/Supplementary Material, and further inquiries can be directed to the corresponding author.

## Supplementary Materials

The following supporting information can be downloaded at: https://www.mdpi.com/article/10.3390/pharmaceutics16111414/s1, Supplementary Data S1: Dataset of Included Studies on Virtual Bioequivalence.

## References

[1] Sowmya C., Abrar H., Prakaash K. (2023). Virtual Bioequivalence in Pharmaceuticals: Current Status and Future Prospects. Int. J. Appl. Pharm..

[2] Kollipara S., Martins F.S., Jereb R., Krajcar D., Ahmed T. (2024). Advancing Virtual Bioequivalence for Orally Administered Drug Products: Methodology, Real-World Applications and Future Outlook. Pharmaceuticals.

[3] Chow S.C. (2014). Bioavailability and Bioequivalence in Drug Development. Wiley Interdiscip. Rev. Comput. Stat..

[4] Glerum P.J., Neef C., Burger D.M., Yu Y., Maliepaard M. (2020). Pharmacokinetics and Generic Drug Switching: A Regulator’s View. Clin. Pharmacokinet..

[5] Hirano M., Yamada M., Tanaka T., Koue T., Saito T., Higashimori M., Ochiai H., Yamamoto J., Yaguchi S., Mita S. (2021). Surveys/Research Exploring Japanese Phase I Studies in Global Drug Development: Are They Necessary Prior to Joining Global Clinical Trials?. Clin. Pharmacol. Drug. Dev..

[6] Tian Y., Reichardt B., Dunkler D., Hronsky M., Winkelmayer W.C., Bucsics A., Strohmaier S., Heinze G. (2020). Comparative effectiveness of branded vs. generic versions of antihypertensive, lipid-lowering and hypoglycemic substances: A population-wide cohort study. Sci. Rep..

[7] Kesselheim A.S., Misono A.S., Lee J.L., Stedman M.R., Brookhart M.A., Choudhry N.K., Shrank W.H. (2008). Clinical equivalence of generic and brand-name drugs used in cardiovascular disease: A systematic review and meta-analysis. JAMA.

[8] Papadopoulos D., Karalis V.D. (2024). Introducing an Artificial Neural Network for Virtually Increasing the Sample Size of Bioequivalence Studies. Appl. Sci..

[9] Chen A., Yarmush M.L., Maguire T. (2012). Physiologically based pharmacokinetic models: Integration of in silico approaches with micro cell culture analogues. Curr. Drug Metab..

[10] Zhuang X., Lu C. (2016). PBPK modeling and simulation in drug research and development. Acta Pharm. Sin. B.

[11] Lin L., Wong H. (2017). Predicting Oral Drug Absorption: Mini Review on Physiologically-Based Pharmacokinetic Models. Pharmaceutics.

[12] Jones H.M., Chen Y., Gibson C., Heimbach T., Parrott N., Peters S.A., Snoeys J., Upreti V.V., Zheng M., Hall S.D. (2015). Physiologically based pharmacokinetic modeling in drug discovery and development: A pharmaceutical industry perspective. Clin. Pharmacol. Ther..

[13] Deepika D., Kumar V. (2023). The Role of “Physiologically Based Pharmacokinetic Model (PBPK)” New Approach Methodology (NAM) in Pharmaceuticals and Environmental Chemical Risk Assessment. Int. J. Environ. Res. Public Health.

[14] Rowland M., Peck C., Tucker G. (2011). Physiologically-based pharmacokinetics in drug development and regulatory science. Annu Rev. Pharmacol. Toxicol..

[15] Siebinga H., de Wit-van der Veen B.J., Stokkel M.D.M., Huitema A.D.R., Hendrikx J. (2022). Current use and future potential of (physiologically based) pharmacokinetic modelling of radiopharmaceuticals: A review. Theranostics.

[16] Mackie C., Arora S., Seo P., Moody R., Rege B., Pepin X., Heimbach T., Tannergren C., Mitra A., Suarez-Sharp S. (2024). Physiologically Based Biopharmaceutics Modeling (PBBM): Best Practices for Drug Product Quality, Regulatory and Industry Perspectives: 2023 Workshop Summary Report. Mol. Pharm..

[17] Shebley M., Sandhu P., Emami Riedmaier A., Jamei M., Narayanan R., Patel A., Peters S.A., Reddy V.P., Zheng M., de Zwart L. (2018). Physiologically Based Pharmacokinetic Model Qualification and Reporting Procedures for Regulatory Submissions: A Consortium Perspective. Clin. Pharmacol. Ther..

[18] Sun Z., Zhao N., Zhao X., Wang Z., Liu Z., Cui Y. (2024). Application of physiologically based pharmacokinetic modeling of novel drugs approved by the U.S. food and drug administration. Eur. J. Pharm. Sci..

[19] Manolis E., García-Arieta A., Lindahl A., Kotzagiorgis E., Limberg J., Holte Ø., Paixao P., Versantvoort C., Tshinanu F.M., Blake K. (2023). Using mechanistic models to support development of complex generic drug products: European Medicines Agency perspective. CPT Pharmacomet. Syst. Pharmacol..

[20] Tsakalozou E., Alam K., Babiskin A., Zhao L. (2021). Physiologically-Based Pharmacokinetic Modeling to Support Determination of Bioequivalence for Dermatological Drug Products: Scientific and Regulatory Considerations. Clin. Pharmacol. Ther..

[21] Lee M., Ly H., Möller C.C., Ringel M.S. (2019). Innovation in Regulatory Science Is Meeting Evolution of Clinical Evidence Generation. Clin. Pharmacol. Ther..

[22] Marques L., Costa B., Pereira M., Silva A., Santos J., Saldanha L., Silva I., Magalhães P., Schmidt S., Vale N. (2024). Advancing Precision Medicine: A Review of Innovative In Silico Approaches for Drug Development, Clinical Pharmacology and Personalized Healthcare. Pharmaceutics.

[23] Alomari N., Alhussaini W. (2024). Update on the advances and challenges in bioequivalence testing methods for complex topical generic products. Front. Pharmacol..

[24] Jiang J., Ma X., Ouyang D., Williams R.O. (2022). Emerging Artificial Intelligence (AI) Technologies Used in the Development of Solid Dosage Forms. Pharmaceutics.

[25] Kapustina O., Burmakina P., Gubina N., Serov N., Vinogradov V. (2024). User-friendly and industry-integrated AI for medicinal chemists and pharmaceuticals. Artif. Intell. Chem..

[26] Yu Y., Loi C.M., Hoffman J., Wang D. (2017). Physiologically Based Pharmacokinetic Modeling of Palbociclib. J. Clin. Pharmacol..

[27] Cho C.K., Kang P., Park H.J., Lee Y.J., Bae J.W., Jang C.G., Lee S.Y. (2021). Physiologically based pharmacokinetic (PBPK) modelling of tamsulosin related to *CYP2D6*10* allele. Arch. Pharm. Res..

[28] Chen G., Sun K., Michon I., Barter Z., Neuhoff S., Ghosh L., Ilic K., Song I.H. (2024). Physiologically Based Pharmacokinetic Modeling for Maribavir to Inform Dosing in Drug-Drug Interaction Scenarios with CYP3A4 Inducers and Inhibitors. J. Clin. Pharmacol..

[29] Kim Y.H., Kang P., Cho C.K., Jung E.H., Park H.J., Lee Y.J., Bae J.W., Jang C.G., Lee S.Y. (2021). Physiologically based pharmacokinetic (PBPK) modeling for prediction of celecoxib pharmacokinetics according to CYP2C9 genetic polymorphism. Arch. Pharm. Res..

[30] Fendt R., Hofmann U., Schneider A.R.P., Schaeffeler E., Burghaus R., Yilmaz A., Blank L.M., Kerb R., Lippert J., Schlender J.F. (2021). Data-driven personalization of a physiologically based pharmacokinetic model for caffeine: A systematic assessment. CPT Pharmacomet. Syst. Pharmacol..

[31] Watanabe A., Ishizuka T., Yamada M., Igawa Y., Shimizu T., Ishizuka H. (2022). Physiologically based pharmacokinetic modelling to predict the clinical effect of CYP3A inhibitors/inducers on esaxerenone pharmacokinetics in healthy subjects and subjects with hepatic impairment. Eur. J. Clin. Pharmacol..

[32] Ou Y., Xu Y., Gore L., Harvey R.D., Mita A., Papadopoulos K.P., Wang Z., Cutler R.E., Pinchasik D.E., Tsimberidou A.M. (2019). Physiologically-based pharmacokinetic modelling to predict oprozomib CYP3A drug-drug interaction potential in patients with advanced malignancies. Br. J. Clin. Pharmacol..

[33] Yee K.L., Cabalu T.D., Kuo Y., Fillgrove K.L., Liu Y., Triantafyllou I., McClain S., Dreyer D., Wenning L., Stoch S.A. (2021). Physiologically Based Pharmacokinetic Modeling of Doravirine and Its Major Metabolite to Support Dose Adjustment With Rifabutin. J. Clin. Pharmacol..

[34] Jo H., Pilla Reddy V., Parkinson J., Boulton D.W., Tang W. (2021). Model-Informed Pediatric Dose Selection for Dapagliflozin by Incorporating Developmental Changes. CPT Pharmacomet. Syst. Pharmacol..

[35] Posada M.M., Cannady E.A., Payne C.D., Zhang X., Bacon J.A., Pak Y.A., Higgins J.W., Shahri N., Hall S.D., Hillgren K.M. (2017). Prediction of Transporter-Mediated Drug-Drug Interactions for Baricitinib. Clin. Transl. Sci..

[36] Zane N.R., Thakker D.R. (2014). A physiologically based pharmacokinetic model for voriconazole disposition predicts intestinal first-pass metabolism in children. Clin. Pharmacokinet..

[37] Lang J., Vincent L., Chenel M., Ogungbenro K., Galetin A. (2020). Simultaneous Ivabradine Parent-Metabolite PBPK/PD Modelling Using a Bayesian Estimation Method. AAPS J..

[38] Hanke N., Teifel M., Moj D., Wojtyniak J.G., Britz H., Aicher B., Sindermann H., Ammer N., Lehr T. (2018). A physiologically based pharmacokinetic (PBPK) parent-metabolite model of the chemotherapeutic zoptarelin doxorubicin-integration of in vitro results, Phase I and Phase II data and model application for drug-drug interaction potential analysis. Cancer Chemother. Pharmacol..

[39] Chen Y., Zhou D., Tang W., Zhou W., Al-Huniti N., Masson E. (2018). Physiologically Based Pharmacokinetic Modeling to Evaluate the Systemic Exposure of Gefitinib in CYP2D6 Ultrarapid Metabolizers and Extensive Metabolizers. J. Clin. Pharmacol..

[40] Gajewska M., Blumenstein L., Kourentas A., Mueller-Zsigmondy M., Lorenzo S., Sinn A., Velinova M., Heimbach T. (2020). Physiologically Based Pharmacokinetic Modeling of Oral Absorption, pH, and Food Effect in Healthy Volunteers to Drive Alpelisib Formulation Selection. AAPS J..

[41] Donovan M.D., Abduljalil K., Cryan J.F., Boylan G.B., Griffin B.T. (2018). Application of a physiologically-based pharmacokinetic model for the prediction of bumetanide plasma and brain concentrations in the neonate. Biopharm. Drug Dispos..

[42] Fu Q., Jones H.M., Sun G., Atamas S.P. (2021). A Physiologically Based Pharmacokinetic and Drug-Drug Interaction Model for the CB2 Agonist Lenabasum. Eur. J. Drug Metab. Pharmacokinet..

[43] Bergagnini-Kolev M., Kane K., Templeton I.E., Curran A.K. (2023). Evaluation of the Potential for Drug-Drug Interactions with Inhaled Itraconazole Using Physiologically Based Pharmacokinetic Modelling, Based on Phase 1 Clinical Data. AAPS J..

[44] Nakamaru Y., Emoto C., Shimizu M., Yamazaki H. (2015). Human pharmacokinetic profiling of the dipeptidyl peptidase-IV inhibitor teneligliptin using physiologically based pharmacokinetic modeling. Biopharm. Drug Dispos..

[45] Thompson E.J., Jeong A., Helfer V.E., Shakhnovich V., Edginton A., Balevic S.J., James L.P., Collier D.N., Anand R., Gonzalez D. (2024). Physiologically-based pharmacokinetic modeling of pantoprazole to evaluate the role of CYP2C19 genetic variation and obesity in the pediatric population. CPT Pharmacomet. Syst. Pharmacol..

[46] Wang H.Y., Chen X., Jiang J., Shi J., Hu P. (2016). Evaluating a physiologically based pharmacokinetic model for predicting the pharmacokinetics of midazolam in Chinese after oral administration. Acta Pharmacol. Sin..

[47] Callegari E., Lin J., Tse S., Goosen T.C., Sahasrabudhe V. (2021). Physiologically-Based Pharmacokinetic Modeling of the Drug-Drug Interaction of the UGT Substrate Ertugliflozin Following Co-Administration with the UGT Inhibitor Mefenamic Acid. CPT Pharmacomet. Syst. Pharmacol..

[48] Nakamura T., Toshimoto K., Lee W., Imamura C.K., Tanigawara Y., Sugiyama Y. (2018). Application of PBPK Modeling and Virtual Clinical Study Approaches to Predict the Outcomes of CYP2D6 Genotype-Guided Dosing of Tamoxifen. CPT Pharmacomet. Syst. Pharmacol..

[49] Stader F., Courlet P., Decosterd L.A., Battegay M., Marzolini C. (2021). Physiologically-Based Pharmacokinetic Modeling Combined with Swiss HIV Cohort Study Data Supports No Dose Adjustment of Bictegravir in Elderly Individuals Living With HIV. Clin. Pharmacol. Ther..

[50] Yang R., Lin Y., Chen K., Huang J., Yang S., Yao A., Yang X., Lei D., Xiao J., Yang G. (2024). Establishing Virtual Bioequivalence and Clinically Relevant Specifications for Omeprazole Enteric-Coated Capsules by Incorporating Dissolution Data in PBPK Modeling. AAPS J..

[51] Ojala K., Schilderink R., Nykänen P., van Veen B., Malmström C., Juppo A., Korjamo T. (2020). Predicting the effect of prandial stage and particle size on absorption of ODM-204. Eur. J. Pharm. Biopharm..

[52] Agyemang A., Farrell C., Moore W., Parkin J. (2021). A Physiologically Based Pharmacokinetic Model to Predict Potential Drug-Drug Interactions and Inform Dosing of Acumapimod, an Oral p38 MAPK Inhibitor. CPT Pharmacomet. Syst. Pharmacol..

[53] Chen Y., Mao J., Hop C.E. (2015). Physiologically based pharmacokinetic modeling to predict drug-drug interactions involving inhibitory metabolite: A case study of amiodarone. Drug Metab. Dispos..

[54] Litou C., Patel N., Turner D.B., Kostewicz E., Kuentz M., Box K.J., Dressman J. (2019). Combining biorelevant in vitro and in silico tools to simulate and better understand the in vivo performance of a nano-sized formulation of aprepitant in the fasted and fed states. Eur. J. Pharm. Sci..

[55] Einolf H.J., Zhou J., Won C., Wang L., Rebello S. (2017). A Physiologically-Based Pharmacokinetic Modeling Approach To Predict Drug-Drug Interactions of Sonidegib (LDE225) with Perpetrators of CYP3A in Cancer Patients. Drug Metab. Dispos..

[56] Yamazaki S., Johnson T.R., Smith B.J. (2015). Prediction of Drug-Drug Interactions with Crizotinib as the CYP3A Substrate Using a Physiologically Based Pharmacokinetic Model. Drug Metab. Dispos..

[57] Riddell K., Patel A., Collins G., Zhou Y., Schramek D., Kremer B.E., Ferron-Brady G. (2021). An Adaptive Physiologically Based Pharmacokinetic-Driven Design to Investigate the Effect of Itraconazole and Rifampicin on the Pharmacokinetics of Molibresib (GSK525762) in Healthy Female Volunteers. J. Clin. Pharmacol..

[58] Purohit V., Sagawa K., Hsu H.J., Kushner J., Dowty M.E., Tse S., Lin J., Blanchard A., Mukherjee A., Le V. (2024). Integrating Clinical Variability into PBPK Models for Virtual Bioequivalence of Single and Multiple Doses of Tofacitinib Modified-Release Dosage Form. Clin. Pharmacol. Ther..

[59] Dennison T.J., Smith J.C., Badhan R.K., Mohammed A.R. (2017). Fixed-dose combination orally disintegrating tablets to treat cardiovascular disease: Formulation, in vitro characterization and physiologically based pharmacokinetic modeling to assess bioavailability. Drug Des. Devel. Ther..

[60] Knöchel J., Panduga V., Nelander K., Heijer M., Lindstedt E.L., Ali H., Aurell M., Ödesjö H., Forte P., Connolly K. (2024). A drug-drug interaction study and physiologically based pharmacokinetic modelling to assess the effect of an oral 5-lipoxygenase activating protein inhibitor on the pharmacokinetics of oral midazolam. Br. J. Clin. Pharmacol..

[61] Freise K.J., Shebley M., Salem A.H. (2017). Quantitative Prediction of the Effect of CYP3A Inhibitors and Inducers on Venetoclax Pharmacokinetics Using a Physiologically Based Pharmacokinetic Model. J. Clin. Pharmacol..

[62] Zhang M., Zhang S., Wang L., Zhang Z., Hu Q., Liu D. (2024). Key Factors for Improving Predictive Accuracy and Avoiding Overparameterization of the PBPK Absorption Model in Food Effect Studies of Weakly Basic Water-Insoluble Compounds in Immediate Release Formulations. Pharmaceutics.

[63] Ding S., Wang L., Xie L., Zhou S., Chen J., Zhao Y., Deng W., Liu Y., Zhang H., Shao F. (2020). Bioequivalence Study of 2 Formulations of Rivaroxaban, a Narrow-Therapeutic-Index Drug, in Healthy Chinese Subjects Under Fasting and Fed Conditions. Clin. Pharmacol. Drug. Dev..

[64] Wang J., Zhang H., Wang R., Cai Y. (2021). Pharmacokinetics, Bioequivalence and Safety Evaluation of Two Ticagrelor Tablets Under Fasting and Fed Conditions in Healthy Chinese Subjects. Drug Des. Devel. Ther..

[65] Boetsch C., Parrott N., Fowler S., Poirier A., Hainzl D., Banken L., Martin-Facklam M., Hofmann C. (2016). Effects of Cytochrome P450 3A4 Inhibitors-Ketoconazole and Erythromycin-on Bitopertin Pharmacokinetics and Comparison with Physiologically Based Modelling Predictions. Clin. Pharmacokinet..

[66] Katsube T., Inoue Y., Fukuhara T., Kano T., Wajima T. (2020). Evaluation of drug-drug interaction of lusutrombopag, a thrombopoietin receptor agonist, via metabolic enzymes and transporters. Eur. J. Clin. Pharmacol..

[67] Andreas C.J., Pepin X., Markopoulos C., Vertzoni M., Reppas C., Dressman J.B. (2017). Mechanistic investigation of the negative food effect of modified release zolpidem. Eur. J. Pharm. Sci..

[68] Post T.M., Gerrits M., Kerbusch T., de Greef R. (2016). Prediction of nomegestrol acetate pharmacokinetics in healthy female adolescents and adults by whole-body physiology-based pharmacokinetic modelling and clinical validation. Contraception.

[69] Li J., Chen J., Kanamaluru V., Gaemers S.J.M., Peterschmitt M.J., Hou A.W., Xue Y., Turpault S., Rudin D. (2020). Impact of hepatic and renal impairment on the pharmacokinetics and tolerability of eliglustat therapy for Gaucher disease type 1. Mol. Genet. Metab..

[70] Samant T.S., Dhuria S., Lu Y., Laisney M., Yang S., Grandeury A., Mueller-Zsigmondy M., Umehara K., Huth F., Miller M. (2018). Ribociclib Bioavailability Is Not Affected by Gastric pH Changes or Food Intake: In Silico and Clinical Evaluations. Clin. Pharmacol. Ther..

[71] Morcos P.N., Schlender J., Burghaus R., Moss J., Lloyd A., Childs B.H., Macy M.E., Reid J.M., Chung J., Garmann D. (2023). Model-informed approach to support pediatric dosing for the pan-PI3K inhibitor copanlisib in children and adolescents with relapsed/refractory solid tumors. Clin. Transl. Sci..

[72] Traver E., Rodríguez-Pascau L., Meya U., Pina G., Pascual S., Poli S., Eckland D., van de Wetering J., Ke A., Lindauer A. (2024). Clinical pharmacokinetics of leriglitazone and a translational approach using PBPK modeling to guide the selection of the starting dose in children. CPT Pharmacomet. Syst. Pharmacol..

[73] Tsamandouras N., Dickinson G., Guo Y., Hall S., Rostami-Hodjegan A., Galetin A., Aarons L. (2015). Development and Application of a Mechanistic Pharmacokinetic Model for Simvastatin and its Active Metabolite Simvastatin Acid Using an Integrated Population PBPK Approach. Pharm. Res..

[74] Salerno S.N., Edginton A., Cohen-Wolkowiez M., Hornik C.P., Watt K.M., Jamieson B.D., Gonzalez D. (2017). Development of an Adult Physiologically Based Pharmacokinetic Model of Solithromycin in Plasma and Epithelial Lining Fluid. CPT Pharmacomet. Syst. Pharmacol..

[75] Venuto C., Simuni T., Fiske B., Coffey C., Matthews H., Wyse R., Brundin P., Simon D., Schwarzschild M., Weiner D. (2020). Physiologically-based pharmacokinetic modeling of nilotinib to determine serum, cerebrospinal fluid, and brain exposures. Mov. Disord..

[76] Rhee S.J., Lee H.A., Lee S., Kim E., Jeon I., Song I.S., Yu K.S. (2018). Physiologically Based Pharmacokinetic Modeling of Fimasartan, Amlodipine, and Hydrochlorothiazide for the Investigation of Drug-Drug Interaction Potentials. Pharm. Res..

[77] Hwang S., Lee Y., Jang Y., Cho J.Y., Yoon S., Chung J.Y. (2024). Comprehensive Evaluation of OATP- and BCRP-Mediated Drug-Drug Interactions of Methotrexate Using Physiologically-Based Pharmacokinetic Modeling. Clin. Pharmacol. Ther..

[78] Samant T.S., Huth F., Umehara K., Schiller H., Dhuria S.V., Elmeliegy M., Miller M., Chakraborty A., Heimbach T., He H. (2020). Ribociclib Drug-Drug Interactions: Clinical Evaluations and Physiologically-Based Pharmacokinetic Modeling to Guide Drug Labeling. Clin. Pharmacol. Ther..

[79] Chen B., Zhou D., Wei H., Yotvat M., Zhou L., Cheung J., Sarvaria N., Lai R., Sharma S., Vishwanathan K. (2022). Acalabrutinib CYP3A-mediated drug-drug interactions: Clinical evaluations and physiologically based pharmacokinetic modelling to inform dose adjustment strategy. Br. J. Clin. Pharmacol..

[80] Djebli N., Fabre D., Boulenc X., Fabre G., Sultan E., Hurbin F. (2015). Physiologically based pharmacokinetic modeling for sequential metabolism: Effect of CYP2C19 genetic polymorphism on clopidogrel and clopidogrel active metabolite pharmacokinetics. Drug Metab. Dispos..

[81] Xiao Q., Tang L., Xu R., Qian W., Yang J. (2015). Physiologically based pharmacokinetics model predicts the lack of inhibition by repaglinide on the metabolism of pioglitazone. Biopharm. Drug Dispos..

[82] Chen J., Ruan Z., Lou H., Yang D., Shao R., Xu Y., Hu X., Jiang B. (2022). First-in-human study to investigate the safety and pharmacokinetics of salvianolic acid A and pharmacokinetic simulation using a physiologically based pharmacokinetic model. Front. Pharmacol..

[83] Kaur N., Thakur P.S., Shete G., Gangwal R., Sangamwar A.T., Bansal A.K. (2020). Understanding the Oral Absorption of Irbesartan Using Biorelevant Dissolution Testing and PBPK Modeling. AAPS PharmSciTech.

[84] Jamei M., Marciniak S., Feng K., Barnett A., Tucker G., Rostami-Hodjegan A. (2009). The Simcyp population-based ADME simulator. Expert Opin. Drug Metab. Toxicol..

[85] Aithal P.A., Aithal S., Aithal S. (2022). Case Study on Certara’s Simcyp PBPK Simulator to Eliminate Lengthy Clinical Trials. Int. J. Health Sci. Pharm. (IJHSP).

[86] Gufford B.T., Barr J.T., González-Pérez V., Layton M.E., White J.R., Oberlies N.H., Paine M.F. (2015). Quantitative prediction and clinical evaluation of an unexplored herb-drug interaction mechanism in healthy volunteers. CPT Pharmacomet. Syst. Pharmacol..

[87] Demeester C., Robins D., Edwina A.E., Tournoy J., Augustijns P., Ince I., Lehmann A., Vertzoni M., Schlender J.F. (2023). Physiologically based pharmacokinetic (PBPK) modelling of oral drug absorption in older adults—An AGePOP review. Eur. J. Pharm. Sci..

[88] Jamei M., Turner D., Yang J., Neuhoff S., Polak S., Rostami-Hodjegan A., Tucker G. (2009). Population-based mechanistic prediction of oral drug absorption. AAPS J..

[89] Cristofoletti R., Dressman J. (2014). Use of Physiologically Based Pharmacokinetic Models Coupled with Pharmacodynamic Models to Assess the Clinical Relevance of Current Bioequivalence Criteria for Generic Drug Products Containing Ibuprofen. J. Pharm. Sci..

[90] Rajput A.J., Aldibani H.K.A., Rostami-Hodjegan A. (2023). In-depth analysis of patterns in selection of different physiologically based pharmacokinetic modeling tools: Part I—Applications and rationale behind the use of open source-code software. Biopharm Drug Dispos..

[91] Darwich A.S., Ogungbenro K., Vinks A.A., Powell J.R., Reny J.L., Marsousi N., Daali Y., Fairman D., Cook J., Lesko L.J. (2017). Why has model-informed precision dosing not yet become common clinical reality? lessons from the past and a roadmap for the future. Clin. Pharmacol. Ther..

[92] Sager J.E., Yu J., Ragueneau-Majlessi I., Isoherranen N. (2015). Physiologically Based Pharmacokinetic (PBPK) Modeling and Simulation Approaches: A Systematic Review of Published Models, Applications, and Model Verification. Drug Metab. Dispos..

[93] Willmann S., Höhn K., Edginton A., Sevestre M., Solodenko J., Weiss W., Lippert J., Schmitt W. (2007). Development of a physiology-based whole-body population model for assessing the influence of individual variability on the pharmacokinetics of drugs. J. Pharmacokinet. Pharmacodyn..

[94] Schmitt W., Willmann S. (2004). Physiology-based pharmacokinetic modeling: Ready to be used. Drug Discov. Today: Technol..

[95] Agoram B., Woltosz W.S., Bolger M.B. (2001). Predicting the impact of physiological and biochemical processes on oral drug bioavailability. Adv. Drug Deliv. Rev..

[96] Bolger M.B., Lukacova V., Woltosz W.S. (2009). Simulations of the nonlinear dose dependence for substrates of influx and efflux transporters in the human intestine. AAPS J..

[97] Luo T., Wang L., Ruan Z., Lou H., Yang D., Wang Z., Zhao P., Jiang B. (2024). Physiologically based absorption modeling to predict the bioequivalence of two apixaban formulations. Clin. Transl. Sci..

[98] Loisios-Konstantinidis I., Cristofoletti R., Fotaki N., Turner D., Dressman J. (2019). Establishing virtual bioequivalence and clinically relevant specifications using in vitro biorelevant dissolution testing and physiologically-based population pharmacokinetic modeling. Case example: Naproxen. Eur. J. Pharm. Sci..

[99] Dermawan D., Bahtiar R., Sofian F.F. (2019). Implementation of Green Supply Chain Management (GSCM) in the pharmaceutical industry in Indonesia: Feasibility analysis and case studies. J. Ilm. Farm..

[100] Pepin X.J.H., Parrott N., Dressman J., Delvadia P., Mitra A., Zhang X., Babiskin A., Kolhatkar V., Suarez-Sharp S. (2021). Current State and Future Expectations of Translational Modeling Strategies to Support Drug Product Development, Manufacturing Changes and Controls: A Workshop Summary Report. J. Pharm. Sci..

[101] Darwich A.S., Ogungbenro K., Hatley O.J., Rostami-Hodjegan A. (2017). Role of pharmacokinetic modeling and simulation in precision dosing of anticancer drugs. Transl. Cancer Res..

[102] Abla N., Howgate E., Rowland-Yeo K., Dickins M., Bergagnini-Kolev M.C., Chen K.F., McFeely S., Bonner J.J., Santos L.G.A., Gobeau N. (2023). Development and application of a PBPK modeling strategy to support antimalarial drug development. CPT Pharmacomet. Syst. Pharmacol..

[103] Khalil F., Läer S. (2011). Physiologically Based Pharmacokinetic Modeling: Methodology, Applications, and Limitations with a Focus on Its Role in Pediatric Drug Development. BioMed Res. Int..

[104] Vora L.K., Gholap A.D., Jetha K., Thakur R.R.S., Solanki H.K., Chavda V.P. (2023). Artificial Intelligence in Pharmaceutical Technology and Drug Delivery Design. Pharmaceutics.

[105] Yadav S., Singh A., Singhal R., Yadav J.P. (2024). Revolutionizing drug discovery: The impact of artificial intelligence on advancements in pharmacology and the pharmaceutical industry. Intell. Pharm..

[106] Dagenais S., Russo L., Madsen A., Webster J., Becnel L. (2022). Use of Real-World Evidence to Drive Drug Development Strategy and Inform Clinical Trial Design. Clin. Pharmacol. Ther..

